# 

**Published:** 1864-10

**Authors:** 


					Content a.
ORIGINAL COMMUNICATIONS
.153
Gun Shot Wounds?Army of .Northern Virginia,
bv F. Wr-
rel, Sn*geon and Insp-c'o." 0' Capitals.
Two Cases of
Gun-Shot Wounds, j r;,,:rr . ?. Depression of Skull, Re-
sulting in -Bpi'epsr: TrepaBVlhqf: and ,"-e R; marks on
tbe Nature ana Flight of 3b,Us. Spherical and Corneal,
bv
B. Roomer, Sui'g: r>3 , A. C. S.
Rcsfi*: tktt of Clavicle,
reported by R. L. Johnseo, Asaisl&ul Surgeon P A C S.
i be Relations of the PfcriosUum to Osteogenesis,
by is.
S. Oaillard, Surgeon anti Modical Inspector.
Gen-Shot
Wound of the Chest treated bv Hermetically Sealing.
ported by P F Browne, Surgeon i.>- Charts First Oi is :n
Ohimbortr.o Hospital.
CHRONICLE OF MEDICAL SCIENCE.
If-4
Splint lor Compound Fracture,
by S. Stacy Sbipton, M. D.
Ex-
tracts from a Lecture on the Laryngoscope,
by Geo. John-
?301), M P
Embolism?Sudden Death.
HarveY,
JbiUsta-
chian Tube and Otoscopy.
Oio'era.
Lithotomy Operations.
OBITUARY
S rgeon John B. Fontaine.
174
INTERESTING ANALYSES OF ARTICLES IN FOREIGN
JOURNALS.
Addifon's Disease. Sir Be-j^tnin Brodie on Homcepathj. Dia-
b?lec: V r W G. Carter. TetHnui of Fourteen Months Du-
ration. TobfiO'O. Chs of Double litems with S;mn ti-
neous Gelation. Nicotine loun<i ia ibe Vi&cera, ol a
Snuff-Taker.
174

				

